# Anti-Renal Fibrotic Effect of Exercise Training in Hypertension

**DOI:** 10.3390/ijms19020613

**Published:** 2018-02-20

**Authors:** Chiu-Ching Huang, Yi-Yuan Lin, Ai-Lun Yang, Tang-Wei Kuo, Chia-Hua Kuo, Shin-Da Lee

**Affiliations:** 1 Kidney Institute and Division of Nephrology, Department of Internal Medicine, China Medical University Hospital, Taichung 40402, Taiwan; cch@mail.cmuh.org.tw; 2 College of Medicine, China Medical University, Taichung 40402, Taiwan; 3 Graduate Program of Translational Medicine, China Medical University, Taichung 40402, Taiwan; 4 Graduate Institute of Clinical Medical Science, China Medical University, Taichung 40402, Taiwan; charlet8116@gmail.com; 5 Department of Sports Sciences, University of Taipei, Taipei 11153, Taiwan; yangailun@gmail.com (A.-L.Y.); kuochiahua@gmail.com (C.-H.K.); 6 Department of Physical Therapy, Graduate Institute of Rehabilitation Science, China Medical University, Taichung, Taichung 40402, Taiwan; goddess77929@gmail.com; 7 Department of Occupational Therapy, Asia University, Taichung 41354, Taiwan; 8 School of Rehabilitation Science, Shanghai University of TCM, Shanghai 201203, China

**Keywords:** fibrosis, hypertension, inflammation, treadmill training

## Abstract

The purpose of this study was to evaluate the effects of exercise training on renal fibrosis in hypertensive rats. Masson’s trichrome staining and Western blotting were performed on the excised renal cortex from sixteen male spontaneously hypertensive rats (SHR), which were randomly divided into either a sedentary hypertensive group (SHR) or exercise hypertensive group (SHR-EX, running on an exercise treadmill for 60 min/day, 5 sessions/week, for 12 weeks), and from eight male Wistar-Kyoto rats which served as a sedentary normotensive group (WKY). The systolic blood pressure (SBP) and renal fibrosis in hypertensive rats improved after exercise training. The inflammatory-related protein levels of interleukin-6 (IL-6) and cyclooxygenase-2 (COX-2), as well as the fibrotic-related protein levels of transforming growth factor-beta (TGF-β), phospho-Smad2/3 (p-Smad2/3), connective tissue growth factor (CTGF), matrix metalloproteinase-9 (MMP-9), and matrix metalloproteinase-2 (MMP-2) were decreased in the SHR-EX group when compared with the SHR group. Exercise training suppressed the hypertension-induced renal cortical inflammatory and fibrotic pathways in hypertensive rat models. These findings might indicate a new therapeutic effect for exercise training to prevent renal fibrosis in hypertensive nephropathy.

## 1. Introduction

Hypertensive nephropathy is the second leading cause of end-stage renal disease, and its prevalence continues to rise worldwide [1,2]. Substantial evidence supports the notion that elevated blood pressure is a critical risk factor to developing chronic kidney disease and end-stage renal disease, which contribute to the progression of renal fibrosis and eventual renal failure [3,4].

A growing body of evidence supports that elevated inflammation is the most important determinant of fibrosis progression [5,6]. High levels of diverse pro-inflammatory cytokines such as interleukin-6 (IL-6) expression levels were observed consistently in the kidneys collected from chronic kidney disease animal models and human associated hypertensive studies [7,8]. Another study showed that renal cortical expression of cyclooxygenase-2 (COX-2) was upregulated in a salt -induced hypertension diet that may play a role in the pathogenesis of hypertensive renal injury [9]. This indicates that the activation of leukocytes and certain cytokines propagate a state of chronic inflammation in hypertensive nephropathy patients, which likely contributes to the progression of chronic kidney disease and end-stage renal disease [10,11].

Sustained hypertension induces renovascular remodeling by altering extracellular matrix components [12]. Renal fibrosis is characterized by an excess deposition of the extracellular matrix, which is due to an imbalance between synthesis and degradation of collagen [13]. Previous studies showed that transforming growth factor (TGF)-β1 expression favors excessive accumulation of extracellular matrix proteins that characterize glomerular and tubulointerstitial cells in hypertensive rats with moderate renal damage [14]. TGF-β is considered to be an important promoter of fibrogenic pathway activation, which phosphorylates Smad2 and Smad3, and then translocates into the nucleus, leading to excessive accumulation of extracellular matrix components [15]. In addition, the connective tissue growth factor (CTGF) appears to be an important downstream mediator for TGF-β to promote fibrogenic pathway activation [16]. TGF-β can also regulate the matrix metalloproteinases (MMPs) [17]. Several reports indicate that MMP-2 and MMP-9, which are expressed in glomerular injury, control extracellular matrix turnover in kidney disease states [18,19,20]. Moreover, increased renal MMP-2 and MMP-9 activity contributes to renal remodeling of the cortex and medulla in the hypertensive kidney [21].

Exercise training may increase blood flow and delivery of oxygen and nutrients to kidneys [22], and regular exercise has been reported to have benefits for the cardiovascular system such as decreased blood pressure, reduced oxidative stress, reduced renal inflammation, decreased urine protein, and fibrogenesis inhibition, which can improve chronic kidney disease management [22,23,24,25]. Exercise training has various benefits for the protection and treatment of hypertension-related kidney disease or kidney failure [25]. However, the mechanisms of the anti-renal fibrotic effects of exercise training remain unclear. Therefore, this study was designed to investigate the effects of exercise training on hypertension-induced renal damage and fibrosis. We hypothesized that exercise training might suppress renal inflammatory conditions and the TGF-β/CTGF-mediated renal fibrotic pathways in hypertension.

## 2. Results

### 2.1. Physical and Cardiovascular Characteristics

There were no significant differences in whole kidney weight (WKW) and whole kidney weight/tibia length (WKW/TL) among the three groups (Table 1). The systolic blood pressure (SBP), diastolic blood pressure (DBP), and mean arterial pressure (MAP) in the SHR group were higher than in the WKY group. After 12 weeks of exercise training, the final SBP significantly decreased in the SHR-EX group when compared with the SHR group (Table 1).

### 2.2. Body Weight and Blood Pressure Changes

**Figure 1:**
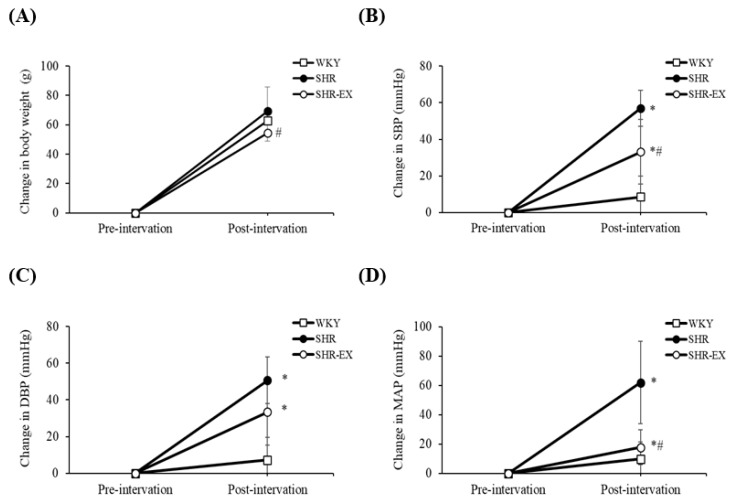
Change in (A) body weight, (B) systolic blood pressure (SBP), (C) diastolic blood pressure (DBP), (D) mean arterial pressure (MAP), pre- to post- exercise training intervention in Wistar-Kyoto rats (WKY), sedentary spontaneously hypertensive rats (SHR), and spontaneously hypertensive rats with exercise training (SHR-EX). Each sub-figure indicates mean values ± SD (*n* = 8 in each group). * *p* < 0.05, significant differences from the WKY group. ^#^ *p* < 0.05, denotes a significant difference between the SHR group and the SHR-EX group.

Pre- to post-intervention, the mean body weight increased in the WKY, SHR, and SHR-EX groups by approximately 63 g (20%), 69 g (25%), and 54 g (18%), respectively (Figure 1A). When compared with the SHR group, the mean body weight was lower in the SHR-EX group by 15 g (−7%) in (Figure 1A). Pre- to post-intervention, the SBP, DBP, and MAP values in the SHR and SHR-EX groups were higher than in the WKY group (Figure 1B–D). When compared with the SHR group, the SBP and MAP values were lower than in the SHR-EX group by 24 mmHg (−35%) and 44 mmHg (−59%), respectively (Figure 1B,D).

### 2.3. Histopathology of Renal Fibrosis Changes

**Figure 2:**
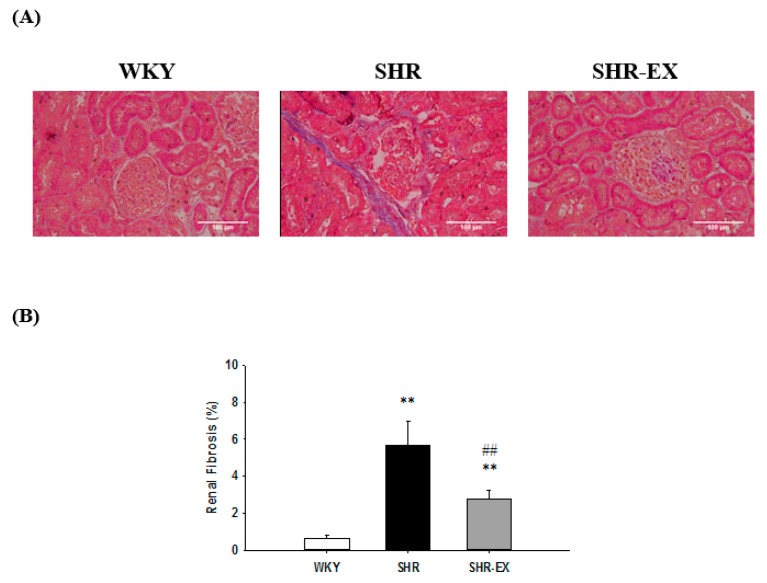
Representative histopathological analysis of kidney sections from the renal cortex in Wistar-Kyoto rats (WKY), sedentary spontaneously hypertensive rats (SHR), and spontaneously hypertensive rats with exercise training (SHR-EX). (**A**) Masson’s trichrome staining (fibrosis: stained blue). The images of the renal cortical architecture were magnified 400×; (**B**) the bar represents the percentage of the blue area to the field area in Masson’s trichrome staining and indicates mean values ± SD (n = 8 in each group). ** *p* < 0.01, denotes a significant difference from the WKY group. ^##^ *p* < 0.01, denotes a significant difference between the SHR group and the SHR-EX group.

To investigate whether there were changes in renal fibrosis after exercise training, we performed a histopathological analysis of the renal cortical slices with Masson’s trichrome staining in kidneys from the WKY, SHR, and SHR-EX groups. We observed that the renal cortex in the SHR group exhibited a much larger area of fibrosis when compared with the WKY group, whereas the renal cortex showed a significant decrease in the SHR-EX group when compared with the SHR group (Figure 2A,B).

### 2.4. Components of Renal Inflammatory Pathway

**Figure 3:**
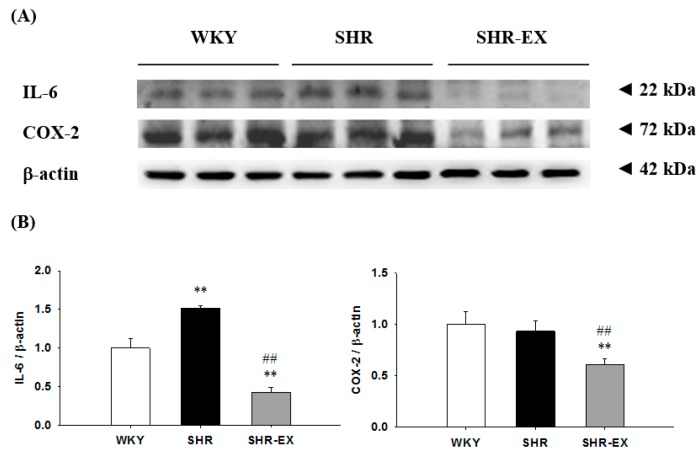
(**A**) The representative protein products of interleukin-6 (IL-6) and cyclooxygenase-2 (COX-2) extracted from the renal cortex of excised kidneys of Wistar-Kyoto rats (WKY), sedentary spontaneously hypertensive rats (SHR), and spontaneously hypertensive rats with exercise training (SHR-EX), as measured by Western blotting analysis; (**B**) the bars represent the relative protein quantification of IL-6 and COX-2 on the basis of β-actin and indicate mean values ± SD (*n* = 8 in each group). ** *p* < 0.01 significant difference from the WKY group. ^##^ *p* < 0.01 significant difference between the SHR group and SHR-EX group.

To investigate the components of the renal inflammatory signaling pathway in hypertensive models with exercise training, the protein levels of interleukin-6 (IL-6) and cyclooxygenase-2 (COX-2) in the renal cortex were excised from the WKY, SHR, and SHR-EX groups. When compared with the WKY group, the protein levels of IL-6 were significantly increased, but the protein levels of COX-2 did not demonstrate a significant difference in the SHR group (Figure 3), whereas they were significantly decreased in the SHR-EX group when compared with the SHR group (Figure 3).

### 2.5. Components of Renal Fibrotic Pathway

**Figure 4:**
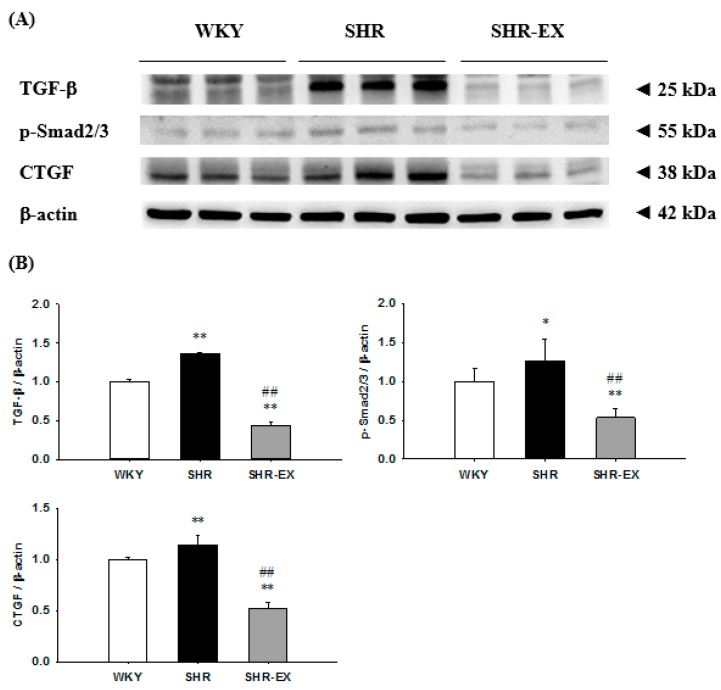
(**A**) The representative protein products of transforming growth factor-beta (TGF-β), phospho-Smad2/3 (p-Smad2/3), and connective tissue growth factor (CTGF) extracted from the renal cortex of excised kidneys of Wistar-Kyoto rats (WKY), sedentary spontaneously hypertensive rats (SHR), and spontaneously hypertensive rats with exercise training (SHR-EX), as measured by Western blotting analysis; (**B**) the bars represent the relative protein quantification of TGF-β, p-Smad2/3, and CTGF on the basis of β-actin and indicate mean values ± SD (n = 8 in each group). * *p* < 0.05 and ** *p* < 0.01 significant difference compared with WKY. ^##^ *p* < 0.01 significant difference between the SHR group and SHR-EX group.

To further understand the components of the renal fibrotic signaling pathway in hypertensive models with exercise training, we measured the protein levels of the transforming growth factor-beta (TGF-β), phospho-Smad2/3 (p-Smad2/3), and connective tissue growth factor (CTGF) in the renal cortex excised from the WKY, SHR, and SHR-EX groups. When compared with the WKY group, the fibrotic protein levels of TGF-β, p-Smad2/3, and CTGF were significantly increased in the SHR group (Figure 4). The protein levels of TGF-β, p-Smad2/3, and CTGF were significantly decreased in the SHR-EX group when compared with the SHR group (Figure 4).

### 2.6. Components of Renal MMPs

**Figure 5:**
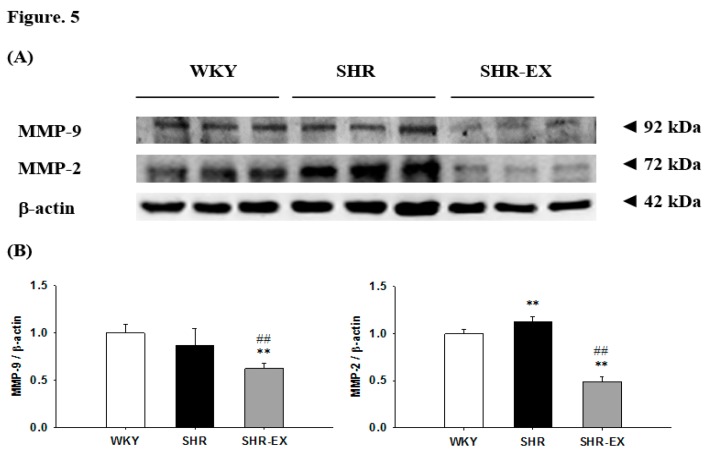
(**A**) The representative protein products of matrix metalloproteinase-9 (MMP-9) and matrix metalloproteinase-2 (MMP-2) extracted from the renal cortex of excised kidneys of Wistar-Kyoto rats (WKY), sedentary spontaneously hypertensive rats (SHR), and spontaneously hypertensive rats with exercise training (SHR-EX), as measured by Western blot analysis; (**B**) the bars represent the relative protein quantification of MMP-9 and MMP-2 on the basis of β-actin and indicate mean values ± SD (n = 8 in each group). ** *p* < 0.01 significant difference compared with WKY. ^##^ *p* < 0.01 significant difference between the SHR group and SHR-EX.

To identify the components of renal MMPs, the protein levels of matrix metalloproteinase-9 (MMP-9) and matrix metalloproteinase-2 (MMP-2) were measured in the excised kidneys of the WKY, SHR, and SHR-EX groups. The protein levels of the MMP-9 did not demonstrate any significant difference, but the protein levels of the MMP-2 were significantly increased in the SHR group when compared with those in the WKY group (Figure 5), whereas they were significantly decreased in the SHR-EX group when compared with the SHR group (Figure 5).

## 3. Discussion

**Figure 6:**
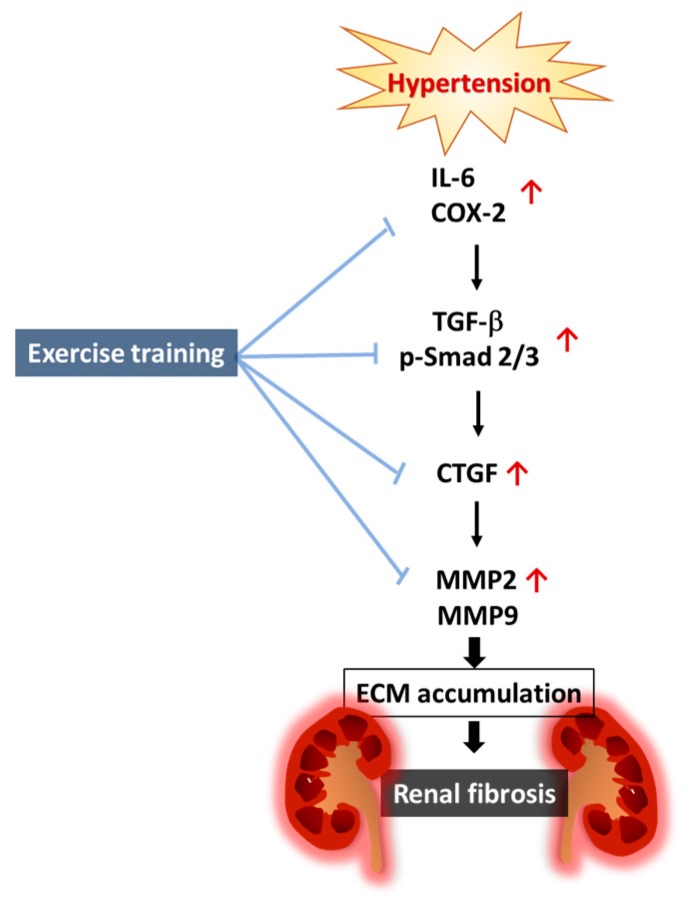
Proposed hypothesized diagram. Exercise training appears to suppress hypertension-induced renal cortical inflammatory components (IL-6 and COX-2), as well as the renal fibrotic pathway (TGF-β, p-Smad2/3, CTGF, MMP-9, and MMP2) and extracellular matrix (ECM) accumulation in hypertensive rat models.

Our main findings are summarized as follows: (1) Elevated renal fibrosis was observed in hypertension; however, it became less severe by exercise training. (2) The hypertension-enhanced renal inflammatory pathway was less activated after exercise training, with evidence of attenuates in interleukin-6 (IL-6) and cyclooxygenase-2 (COX-2) after exercise training, when compared with the sedentary hypertension. (3) The hypertension-enhanced renal fibrotic pathways were less activated after exercise training, with evidence of attenuates in transforming growth factor-beta (TGF-β), phospho-Smad2/3 (p-Smad2/3), connective tissue growth factor (CTGF), matrix metalloproteinase-9 (MMP-9), and matrix metalloproteinase-2 (MMP-2) after exercise training, when compared with the sedentary hypertension. Our hypothesis proposed that hypertension-enhanced renal inflammatory and fibrotic pathways could be suppressed through exercise intervention (Figure 6).

Clinical and pathological features in patients with hypertensive kidney disease includes renal inflammation, renal tubular atrophy, and renal fibrosis [26]. Substantial fibroblast activation, and excessive production and deposition of extracellular matrix contribute to the destruction of renal parenchyma and progressive loss of kidney function [26]. However, previous studies suggest that exercise training might decrease renal fibrosis area in pregnant hypertensive rats [22,27]. Our results are similar to a previous report that shows that renal interstitial fibrosis increased in hypertensive kidneys and hypertension-enhanced renal interstitial fibrosis improved after exercise training by running on a treadmill for twelve weeks.

Chronic inflammation was an important feature in the initiation and progression of hypertensive kidney disease [10,11]. Notably, both animal and human studies reported that elevated IL-6 expression levels contribute to the pathogenesis of chronic kidney disease in hypertension [7]. Nevertheless, some studies have showed that IL-6 attenuation could attenuate angiotensin II–induced hypertension and kidney injury. Moreover, the angiotensin II receptor blocker can reduce the renal cortex COX-2 expression [9]. In the present study, we found that the hypertension-elevated renal cortex IL-6 and COX-2 were attenuated after twelve weeks of exercise training. Therefore, our findings strongly suggest that exercise training did prevent or attenuate hypertension-enhanced renal inflammation.

The TGF-β/Smad signaling pathway acts as an important role for crosstalk between the inflammatory response and fibrosis, which contributes to renal damage and renal fibrosis [15]. Numerous studies have reported that elevated CTGF is involved in hypertension-enhanced renal fibrosis [28,29]. Moreover, previous studies indicate that MMP-2 and MMP-9 are overexpressed in the glomerulus in kidney disease states [18,19,20]. One study also demonstrated that increased renal MMP-2 and MMP-9 activity contributes to glomerular injury and renal remodeling in hypertensive rats [21]. Similarly, the inhibition of TGF-β signaling activity or CTGF expression also decreases renal tubulointerstitial fibrosis [15,30]. Exercise training is well known to decrease blood pressure and prevent renal fibrosis [25], but the detailed mechanisms remain to be clarified. The current study showed that exercise training suppressed TGF-β, p-Smad2/3, CTGF, MMP2, and MMP9 expression to decrease renal fibrosis. This implies that exercise training improves cardiac fibrosis through downregulation of the TGF-β signaling pathway. Our findings suggest that exercise training improves renal abnormalities in hypertension partially through the downregulation of the TGF-β signaling pathway.

Additionally, many lines of evidence have suggested that the renin-angiotensin system is the primary regulator of intravascular volume and systemic blood pressure [31]. Angiotensin II causes elevated blood pressure through its direct vasoconstrictor action [32]. Moreover, angiotensin II also stimulates extracellular matrix proteins synthesis through the upregulation of TGF-β signaling activity, which may lead to renal fibrosis [33]. One study demonstrated that angiotensin II induces a nicotinamide adenine dinucleotide phosphate-oxidase (NADPH oxidase) dependent generation of reactive oxygen species that further leads to the activation of TGF-β and upregulates its downstream fibrogenic effector CTGF [34]. The angiotensin II-induced TGF-β and CTGF may potently stimulate the extracellular matrix protein synthesis and fibroblast proliferation [35]. However, the mechanism of the angiotensin II-induced inflammatory and fibrotic production in hypertension was not determined in this work and further studies are required to evaluate this mechanism.

### Hypothesized and Clinical Application

Our current findings indicate that exercise training may be an important lifestyle modification to prevent hypertensive renal inflammation and fibrosis. Exercise training might provide one possible mechanism to interrupt renal fibrosis and prevent the development of hypertensive kidney disease. We might further hypothesize that exercise training may be one of several important therapeutic approaches to prevent renal fibrosis in hypertension. Of course, further clinical studies are required to clarify any possible therapeutic application.

## 4. Experimental Section

### 4.1. Animals

Eight male Wistar-Kyoto rats (WKY) and sixteen spontaneously hypertensive rats at 6 weeks old were purchased from the National Laboratory Animal Breeding and Research Center, Taipei, Taiwan. The spontaneously hypertensive rats were divided into a sedentary group (SHR) and an exercise group (SHR-EX). The rats were fed with standard laboratory chow (Lab Diet 5001; PMI Nutrition International, Brentwood, CA, USA) and provided with water ad libitum. They were housed in a temperature-controlled room at 25 °C with a 12-h dark/light cycle. All protocols were handled according to the Institutional Animal Care and Use Committee of the University of Taipei Animal Center (IACUC Approval No: 20100001, date: 27 December 2010), and the principles of laboratory animal care (NIH publication) were followed.

### 4.2. Exercise Training

At thirteen-weeks-old, the SHR-EX group trained on a motor-driven leveled treadmill (Model T408E, Diagnostic & Research Instruments Co., Taoyuan, Taiwan) at a speed of 15 m/min for 20 min on the first day. The running time was extended by 10 min/day until a running time of 60 min/day was reached within the familiarization period of 5 days. During the training period, the training speed of 18 m/min gradually increased by 3 m/min every 2 weeks until 27 m/min was achieved. These animals trained for 60 min/day, 5 days/week, for 12 weeks. The sedentary WKY and SHR groups were placed on the treadmill without any exercise training for the same environmental stimulation. At the end of the experimental period, rats were anaesthetized with 2% isoflurane delivered in oxygen (95% O_2_ and 5% CO_2_), and were sacrificed with minimized suffering.

### 4.3. Resting Blood Pressure and Heart Rate

The systolic/diastolic blood pressure (SBP/DBP), mean arterial pressure (MAP), and heart rate (HR) were measured with five consecutive readings by a tail-cuff pressure meter system (LE5001, Panlab, Wood Dale, IL, USA).

### 4.4. Masson’s Trichrome Staining

The excised kidneys were soaked in 4% formalin, dehydrated through graded alcohols, and embedded in paraffin wax. Paraffin sections 4-µm thick were cut from the paraffin-embedded tissue blocks. The tissue sections were deparaffinized by immersion in xylene for 5 min two times and rehydrated through graded alcohols (1 min each in 100%, 90%, 85%, and 75%). Next, all slices were stained with Masson’s trichrome kit that was soaked in a warmed Bouin’s solution at 60 °C for 45 min and then washed in running tap water for 10 min. All slides were immersed in Weigert’s haematoxylin for 5 min followed then washed in running tap water for 2 min. The slides were stained with acid fuchsin for 15 min and then rinsed in distilled water. Next, the slides were treated with phosphomolybdic acid solution for 10 min and then immediately stained with methyl blue solution for 10 min. Slides were rinsed with distilled water and treated with 1% acetic acid solution for 3 min. Each slide was dehydrated through two changes of alcohol (95%, 95%, 100%, and 100%). Finally, they were soaked in xylene twice. Photomicrographs were obtained using a phase-contrast microscope (400×, Olympus BX43, Tokyo, Japan). The quantification of fibrotic areas (stained blue) and cortex areas (stained red) was performed using Image J analysis software. The fibrosis percentages of the renal cortical areas were obtained by calculating the ratio of the fibrotic area to renal cortical area.

### 4.5. Western Immunoblotting

The excised kidneys were cleaned and frozen. The renal cortical tissue extracts were obtained by homogenizing at 4 °C in a tissue protein extraction reagent (T-PER, Thermo Scientific, Waltham, MA, USA) supplemented with complete protease and phosphatase inhibitors (Roche Applied Science, Mannheim, Germany). The homogenates were sequentially centrifuged at 12,000× *g* for 40 min and the supernatant was collected. Protein concentration of the renal tissue extract supernatant was determined by the Bradford method (Bio-Rad Laboratories, Hercules, CA, USA), using bovine serum albumin (BSA) as a standard. Protein samples (40 μg/lane) were separated on a 12% sodium dodecyl sulfate polyacrylamide gel electrophoresis (SDS-PAGE) with a constant voltage of 75 V. Electrophoresed proteins were transferred to a polyvinylidene difluoride (PVDF) membrane (0.45-μm pore size, Millipore, Bedford, MA, USA) with a transfer apparatus (Bio-red, Hercules, CA, USA). PVDF membranes were incubated in 5% non- fat dried milk in a Tris-buffered saline with Tween 20 (TBST) buffer (25 mM Tris-HCl, 150 mM NaCl, pH 7.6, and 0.1% Tween-20). Primary antibodies including IL-6, COX-2, TGF-β, p-Smad2/3, CTGF, MMP-9, MMP-2 (Santa Cruz Biotechnology, Santa Cruz, CA, USA), and β-actin (Santa Cruz) were diluted to 1:500 in an antibody binding buffer overnight at 4 °C. The immunoblots were washed in a TBST buffer three times for 10 min. This was then followed by incubation with a horseradish peroxidase (HRP)-conjugated second antibody solution (1:5000 dilution, Santa Cruz Biotechnology) for 60 min at room temperature. The immunoblots were then washed in the TBST buffer three times for 10 min. The immunoblotted proteins were visualized using an enhanced chemiluminescence ECL Western Blotting Luminol Reagent (Millipore Corporation, Billerica, MA, USA) and quantified using a Fujifilm LAS-3000 chemiluminescence detection system (Fuji, Tokyo, Japan).

### 4.6. Statistical Analysis

All data of kidney weights, body weight changes, cortical fibrosis percentages, and protein levels were compared between the WKY, SHR, and SHR-EX groups using one-way ANOVA with preplanned contrast comparison with the control group. The WKY and SHR served as the negative control and the positive control, respectively. For all statistical tests, *p* < 0.05 was considered to be significant.

## Figures and Tables

**Table 1:** Cardiac characteristics of WKY, SHR-Sham, and SHR-EX groups.

Parameters/Groups	WKY	SHR	SHR-EX
**Number of animals**	8	8	8
**Kidney Weight Index**			
WKW (g)	1.28 ± 0.08	1.30 ± 0.12	1.41 ± 0.12
WKW (g)/TL (mm)	0.033 ± 0.002	0.036 ± 0.003	0.032 ± 0.014
**Blood pressure**			
initial SBP (mmHg)	123 ± 11	146 ± 8 **	150 ± 11 **
initial DBP (mmHg)	89 ± 9	105 ± 15 *	114 ± 15 **
initial MAP (mmHg)	100 ± 9	119 ± 12 **	126 ± 12 **
final SBP (mmHg)	134 ± 4	203 ± 8 **	183 ± 12 **^##^
final DBP (mmHg)	98 ± 7	157 ± 8 **	148 ± 13 **
final MAP (mmHg)	110 ± 5	172 ± 7 **	159 ± 13 **

Values are means ± standard deviation (SD). Three groups: Wistar-Kyoto rats (WKY), sedentary spontaneously hypertensive rats (SHR), and spontaneously hypertensive rats with exercise training (SHR-EX). WKW, whole kidney weight; TL, tibia length; SBP, systolic blood pressure; DBP, diastolic blood pressure; MAP, mean arterial pressure. * *p* < 0.05, ** *p* < 0.01, significant differences from the WKY group. ^##^ *p* < 0.01, significant differences between SHR-EX and SHR group.
